# Considering tumour volume for motion corrected DWI of colorectal liver metastases increases sensitivity of ADC to detect treatment-induced changes

**DOI:** 10.1038/s41598-019-40565-y

**Published:** 2019-03-07

**Authors:** Ryan Pathak, Jingduo Tian, Neil A. Thacker, David M. Morris, Hossein Ragheb, Charles Saunders, Mark Saunders, Alan Jackson

**Affiliations:** 1 University of Manchester, Wolfson Molecular Imaging Centre, Manchester, UK; 2 The Christie Hospital, NHS Foundation Trust, Manchester, UK; 3 University of St Andrews, St Andrews, Scotland UK; 4 MRC Centre for Inflammation Research, University of Edinburgh, Edinburgh, UK

## Abstract

ADC is a potential post treatment imaging biomarker in colorectal liver metastasis however measurements are affected by respiratory motion. This is compounded by increased statistical uncertainty in ADC measurement with decreasing tumour volume. In this prospective study we applied a retrospective motion correction method to improve the image quality of 15 tumour data sets from 11 patients. We compared repeatability of ADC measurements corrected for motion artefact against non-motion corrected acquisition of the same data set. We then applied an error model that estimated the uncertainty in ADC repeatability measurements therefore taking into consideration tumour volume. Test-retest differences in ADC for each tumour, was scaled to their estimated measurement uncertainty, and 95% confidence limits were calculated, with a null hypothesis that there is no difference between the model distribution and the data. An early post treatment scan (within 7 days of starting treatment) was acquired for 12 tumours from 8 patients. When accounting for both motion artefact and statistical uncertainty due to tumour volumes, the threshold for detecting significant post treatment changes for an individual tumour in this data set, reduced from 30.3% to 1.7% (95% limits of agreement). Applying these constraints, a significant change in ADC (5^th^ and 20^th^ percentiles of the ADC histogram) was observed in 5 patients post treatment. For smaller studies, motion correcting data for small tumour volumes increased statistical efficiency to detect post treatment changes in ADC. Lower percentiles may be more sensitive than mean ADC for colorectal metastases.

## Introduction

The apparent diffusion coefficient (ADC) is calculated from diffusion-weighted magnetic resonance imaging (DWI)^1^. Selected tissue volumes are sensitised to free water diffusion using strong magnetic gradients so that signal is lost at a rate proportional to the rate of Brownian motion along the encoded direction which, in the range detected by conventional clinical DWI sequences, occurs primarily in the extravascular extracellular space (EES)^2^. ADC will therefore be affected by the size and configuration of the EES but is also affected by other factors such as the local macromolecular environment, the presence of necrotic or cystic areas or fibrosis. In oncological applications increases in ADC in response to treatment has been taken to represent decrease in cell density or loss of the diffusion restriction by cell membranes as cells die or apoptose^3^. ADC offers a potential early response biomarker for clinical trials or personalised therapeutic regimes^4^, however measured changes must be reliably attributed to therapeutic response, not to measurement error or noise^5–7^. Accurate ADC measurements would increase confidence in post treatment response and could be combined with other potential markers e.g. lactate dehydrogenase enzyme (LDH) levels^8,9^.

Previous studies in the liver have found post-treatment mean ADC changes in the range of 10 to 30%^10,11^. It is important to avoid misinterpretation of post treatment mean ADC changes by calculating repeatability/reproducibility^12,13^ and establishing either study-specific baseline thresholds or appropriate estimates where test-retest measurements are not possible^14^. Accurate estimation of other metrics from whole tumour 3D histograms could also increase observed post treatment changes. Examples where histogram analysis has been applied include the brain^15,16^, peritoneum^17^ and the liver^18^. In a study of glioma, the 5^th^ percentile was best for differentiating between high and low grade tumour^16^. The 25^th^ percentile was most sensitive to post treatment ADC changes in peritoneal tumours^17^. In a study comparing whole liver ADC with and without colorectal metastatic tumours, the 5^th^ percentile was significantly lower for the diseased group^18^.

ADC is calculated from multiple DWI acquisitions that assume perfect spatial registration between images therefore significant misregistration from motion will affect ADC accuracy. Respiratory triggering or use of navigator echo techniques can mitigate motion effects, improving image quality in terms of SNR, while maintaining stable ADC values, when compared to breath-hold sequences^19,20^ and free breathing acquisitions^21^. There is however conflicting evidence with other studies showing no advantage to navigator triggering^22^ with decrease in reproducibility and ADC stability compared to free breathing^23,24^.

As shown in our previous work, uncertainty in the accurate estimation of ADC due to statistical measurement errors also adversely affects the ability to reliably detect change^25^. The smaller the sample and wider the distribution of ADC voxel values, the greater the statistical uncertainty around the mean ADC estimate. Instability in repeated measures from smaller volumes has been observed with increased coefficient of variance (CoV)^14^, and size dependent improved reproducibility with whole tumour volumes^26^. The CoV for ADC is a group statistic (ratio of the standard deviation and mean ADC for the study group) that allows comparison of ADC reproducibility between studies and requires large cohorts to infer significant differences when comparing matched study groups, rather than for individual tumour ADC changes.

We hypothesised that correcting for motion artefact and accounting for statistical measurement error, factors previously identified that negatively affect accuracy in ADC, would increase our confidence in attributing a post treatment ADC change as due to biological differences rather than measurement error or noise.

## Materials and Methods

### Patients

This single site prospective study was compliant with and approved by the NHS Health Research Authority Research Ethics Committee, United Kingdom, following approval from local Research & Development administrations at The Christie Hospital NHS Trust, Manchester. Formal written informed consent was recorded for each volunteer that participated. Volunteers were recruited from the colorectal oncology clinic and imaged consecutively, as they presented.

Inclusion criteria included; Histological primary colorectal carcinoma, radiological liver metastasis (at least one, minimum volume 1 cm^3^), no ongoing treatment. Exclusion criteria were contraindications to MRI or ongoing treatment. Patients were scanned on two separate occasions within 1–7 days prior to any new treatment commencing. Patients, who were medically fit and did not withdraw consent, were re-scanned within 1 week after commencing chemotherapy (1^st^ or 2^nd^ line regime).

### Image acquisition

Images were acquired on a Philips Achieva 1.5 Tesla scanner. DWI (twice refocused diffusion-encoding scheme) were acquired in two slightly different ways on the same patients, each method labeled A and B (See Table 1 for acquisition parameters). The differences between methods are outlined as follows; for A, 18 images (6 repeats in 3 orthogonal directions) were acquired at each slice position and averaged by the scanner software to form the final axial slice image composite (1 of 20 slices). In B, for each slice, 6 repeated acquisitions in 3 orthogonal directions were acquired individually and transferred to a standalone workstation for retrospective post processed motion correction. Although A data could be synthesized from the raw B data we chose to acquire A as a separate data set to avoid correlated errors in the analysis.

**Table 1:** We acquired A and B data separately, however protocol A can be synthesized from the raw B data.

Acquisition parameters for protocol A and protocol B
B values of 100, 200, 400, 600 s/mm^2^ (3 orthogonal gradient directions)
6 signal averages per image
TR 8000, TE 88
Single shot echo-planar sequence (SS-EPI)
SENSE parallel imaging
Spectral attenuated inversion recovery (SPAIR) fat suppression
5 mm axial slice thickness, 20 slices with no inter-slice gap
FOV of 384 × 384
Bandwidth 1400–1800 Hz per pixel
Pixel size of 1.5 × 1.5 mm
Acquisition matrix 128 × 128
Pixel size of 1.5 × 1.5 mm (therefore voxel volume 11.25 mm^3^)

### Motion correction

**Figure 1:**
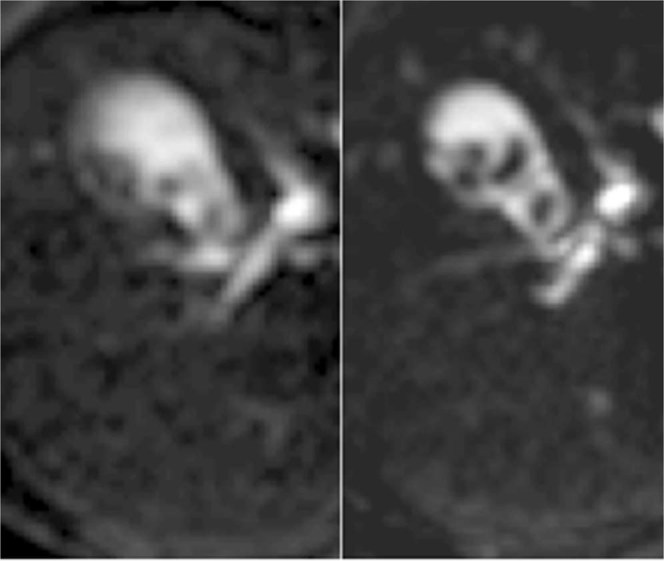
A comparison of part of the liver and gallbladder of a patient with b-100 mm/s^2^ images acquired using standard protocol A (Left) versus acquired and post-processed motion corrected protocol B (Right). Post motion-correction, gallstones become clearly delineated within the high signal bile.

A detailed description of the motion correction method has been published open-access^27^. In brief, a local-rigid alignment (LRA) method designed specifically for use with DWI data was used. A reference slice was chosen (b-100) and split into 4 quadrants with the quadrant containing the tumour used to match adjacent slices. Limits in the degree of allowed movement were set to 3 pixels in x and y directions and 2 slices in the z directions (above and below), based on our observations of typical respiratory motion in liver data. There was little or no observable rotation (less than a pixel at the edge of an ROI). For each reference slice, 2 slices above and 2 below were used for matching. An existing matching algorithm was used for the reference slice against target slice based upon conventional statistics to optimise the cost function (Supplementary Information Appendix 1). The reference slice had 6 repeated acquisitions; therefore, in total there were 30 potential slices to match from when you include the 2 slices above and below for each repeat. The 6 most closely matched from the 30 potential slices were selected, and this process was repeated for each of the 3 orthogonal gradient directions. These 18 slices were then combined to form the composite image for that axial slice position. This was repeated for each axial slice acquired through the liver (20 slices). As the process required 2 slices above and below for each repeated acquisition in a given gradient direction, the top and bottom 2 slice positions could not be used for motion correction. An example of the effects of this method of LRA is given in Fig. 1 (note that the gallstones within the gallbladder become sharper and easier to delineate).

### Image analysis and lesion definition

**Figure 2:**
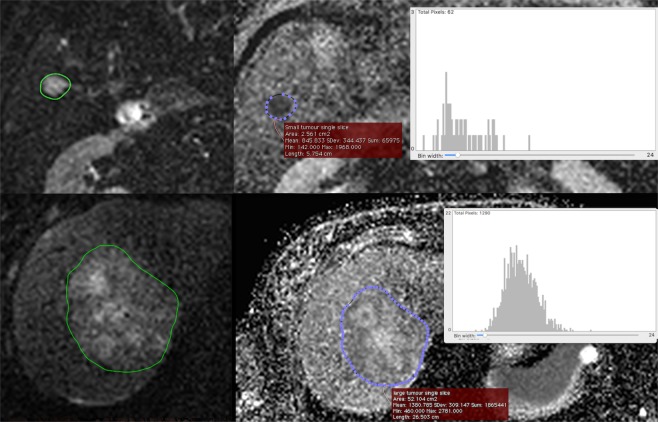
Tumour regions were delineated on b-100 mm/s^2^ diffusion images (left). Histogram analysis was then performed for the corresponding parametric ADC map (right) for whole tumour volumes (single slice represented here). A comparison of ADC distributions for a small tumour (top right) to a large tumour (bottom right) is given as an example. The Y-axis indicates frequency (number of voxels), and the X-axis indicates increasing ADC values (x 10^−5^ mm^2^/s). The mean ADC for that ROI and the standard deviation is given in the red box.

ADC values were estimated by mono-exponential fitting of 4 b-value images (100, 200, 400, 600 s/mm^2^) corrected for high b-value SNR bias^28^ (Supplementary Information Appendix 2). Manual whole tumour ROIs (largest and second largest where available, greater than 1 cm^3^) were delineated from averaged b-100 image slices. The first and last slices through the tumour were excluded to minimise partial volume effects. The process was performed independently for A and B datasets. A manual delineation method was chosen as, in our experience, automated or semi-automated ROI selection methods are less robust in the liver, specifically due to physiological motion and low SNR (Fig. 2). In order to maximise the range of ROI sizes available for the error model (see below), single slice ROIs were also defined within the delineated 3D tumour volume.

### Statistical error model for uncertainty in ADC estimation

A statistical measurement error model estimating ADC uncertainty has been described fully in a previous publication within this journal and is available open access^25^. Briefly, the estimate of a mean or percentile to accurately describe a histogram is dependent on the sample size of the distribution (equivalent to tumour volume in this case). The wider the distribution the larger the error in accurately estimating a given metric and conversely, the larger the sample size and narrower the distribution width, the more precise the estimation will be. Motion artefact, SNR, tumour heterogeneity, and tumour boundary mismatches between test-retest volumes would also be expected to affect distribution width. All of these variables, as well as those we have not considered to affect the statistical accuracy, contribute to measurement error and are accounted for within the error model in terms of the ADC distribution width of an individual tumour.

The difference in ADC histograms between a single slice from a large tumour and small tumour are given as an example in Fig. 2. The larger ROI has a more bell-curve distribution, with a narrower proportional standard deviation, whereas the smaller tumour has a skewed distribution with a much larger proportional standard deviation.

The error model we have applied to this data was originally fitted to ADC calculated from quality assured test-retest tumour volumes (i.e. minimal visible motion artefact) using an error propagation method, to estimate measurement uncertainty for ΔADC% (the percentage change in ADC between baselines).

The suitability of the error model (S) to describe the distribution of this data set with (B) and without (A) motion correction was tested using Chi-squared (χ^2^) goodness of fit (see below). Where the data was of sufficient quality to describe the inverse relationship between tumour volume and statistical measurement uncertainty^25^, the error model could be appropriately applied in order to standardise/scale individual tumours to their level of measurement uncertainty.

### Sample size

Tumour response to chemotherapy agents that may have a variety of mechanisms of action can be heterogeneous due to micro-environmental or genetic factors, as well as geographical variations, i.e. spatial heterogeneity^29–31^. In this study, in order to reach the target sample size of 15, each lesion was treated as an independent entity for repeatability analysis and in the assessment of early post treatment change.

### Repeatability statistics

Histogram analysis of repeatability and ΔADC% LoA for individual tumours, were calculated with a 5% level of significance. The group coefficient of variance (CoV) was also calculated (for A, B and S) to compare this data set with other published studies. CoV is calculated as the ratio (%) of the standard deviation of the group (difference between test and retest absolute ADC) and the average absolute ADC measurement (10^−5^ mm^2^/s). Tumour volumes were also compared at each visit (A vs. B for test and A vs. B for retest) and between visits (volume repeatability) in order to assess the stability of volume delineation. The definitions and formulas required for calculating 95% LoA and CoV can be found in the reference by *Winfield et al*.^14^. All repeatability statistical formulas and subsequent calculations were constructed with analysis performed using Microsoft Excel 2011 (OS X).

This study compared repeatability of ΔADC% histogram metrics between a post-acquisition processing method of motion correction and a non-motion corrected acquisition for the same tumour data. Where the data was deemed suitable (Chi-squared (χ^2^) goodness of fit) for application of an error model that takes into consideration tumour volume, individual tumour ΔADC% was scaled to the estimated level of measurement uncertainty in the measurement. We have assigned this process and the outcomes as “method S” for “standardisation/scaling”.

The χ^2^ goodness to fit tests the independence of two distributions, in this case the estimated uncertainty in individual tumour ΔADC% values for this study data set (A and B) against the distribution of uncertainty for the original error model. If the two are found to be independent then the error model parameters cannot be used to standardise to the level of uncertainty in ΔADC% measurements, implying that there are other factors dominating over tumour size (e.g. motion) and influencing the variability between test and retest measurements.

### Observations of ΔADC% as a marker for early response to treatment

The 95% LoA for ΔADC% between test and retest acquisitions was used to determine the threshold that would be required for an observed post treatment response to reach statistical significance for this cohort. Post treatment ADC was compared to the retest rather than test ADC values in order to minimise any potential biological changes prior to treatment and determine whether any of the three approaches could observe a post treatment ΔADC% response with statistical significance. The pre-treatment ADC values (re test) for patients with a post treatment acquisition are highlighted in bold (Table 3).

**Table 2:** Lesion volumes and ADC values for protocol A and B.

Lesion	Test volume		Retest volume		Volume repeatability		Test ADC		Retest ADC	
	A	B	A	B	ΔVOL% A	ΔVOL% B	A	B	A	B
1	19.8	21.8	21.8	22.6	1.9	0.8	111.9	102.6	**105.5**	**105.6**
2	88.3	84.3	86.4	86.3	1.9	2.0	189.9	189.0	**181.3**	**182.0**
3	105.1	104.6	114.7	112.7	9.6	8.0	115.5	114.4	**114.3**	**111.0**
4	4.4	5.1	6.1	6.0	1.7	0.9	95.7	120.2	**105.3**	**113.8**
5	13.6	13.2	15.1	13.6	1.5	0.4	202.2	187.4	173.7	193.5
6	53.7	47.5	52.2	43.5	1.5	3.9	128.0	122.1	**125.3**	**119.8**
7	7.1	7.4	8.0	7.7	0.9	0.3	107.1	107.3	**114.8**	**113.0**
8	3.5	4.0	2.6	3.5	0.9	0.5	108.9	108.2	**106.0**	**108.1**
9	90.2	86.3	88.3	83.4	1.9	2.9	122.6	115.9	110.0	107.0
10	1.6	1.2	1.1	1.5	0.4	0.3	67.9	78.9	94.0	78.3
11	115.7	114.6	124.4	123.5	8.7	8.9	130.0	120.1	**125.3**	**122.9**
12	8.7	8.5	10.0	8.3	1.3	0.2	116.5	109.7	**95.7**	**97.0**
13	7.9	8.4	4.6	7.8	3.3	0.6	121.9	123.5	**79.8**	**121.1**
14	5.4	5.0	5.4	4.8	0.0	0.2	120.2	124.0	**127.9**	**124.9**
15	9.3	8.9	9.5	8.6	0.2	0.3	126.4	114.2	**122.4**	**116.0**
CoV	4.5%		5.6%		7.2%	6.8%	5.6%		7.7%	

Volume delineation (cm^3^) and calculated mean ADC (mm^2^/s) was similar between standard (A) and motion corrected (B) methods for both the test and retest baseline acquisitions of the same tumour (CoV of 4.5% and 5.6% for volume, 5.6% and 7.7% for ADC). Volumes were repeatable for both A and B with 7.2% and 6.8% percentage change in volume between test and retest (ΔVOL%). Average mean ADC was 121 × 10^−5^ mm^2^/s for A and 122 × 10^−5^ mm^2^/s after motion correction. The bold values in the “Retest” column indicate the pre-treatment tumours used for post treatment response.

**Table 3:** Suitability of the statistical error model.

Protocol	Histogram	CCV	DF	P-Value	Null hypothesis
A	5^th^ percentile	82.86	14	<0.00001	Rejected
A	20^th^ percentile	69.51	14	<0.00001	Rejected
A	Median	56.58	14	<0.00001	Rejected
A	Mean	63.41	14	<0.00001	Rejected
A	95^th^ percentile	95.73	14	<0.00001	Rejected
B	5^th^ percentile	19.5	14	0.145	Accepted
B	20^th^ percentile	14.36	14	0.423	Accepted
B	Median	13.59	14	0.481	Accepted
B	Mean	12.82	14	0.541	Accepted
B	95^th^ percentile	25.39	14	0.031	Rejected

The goodness of fit between the distribution of estimates of uncertainty for each ΔADC% measurement and the statistical error model was assessed using χ^2^ distributions testing (non-motion corrected A vs. motion corrected B). If the null hypothesis of no significant difference between distributions was accepted, then ΔADC% for each tumour could be safely standardised for statistical measurement uncertainty. The critical χ^2^ value (CCV) and degrees of freedom (DF) are displayed.

### Observations of ΔADC% against post treatment LDH trends

Serial serum LDH (U/L) was taken at two weekly intervals as part of routine care and used as a biomarker of disease response. The percentage change in LDH (ΔLDH%) from pre treatment to 3 months post treatment was calculated to observe any association with significant changes in ADC. A Pearson Correlation Coefficient was calculated to observe any significant correlation between ΔADC% and ΔLDH%.

## Results

11 patients (1 female) were recruited for test-retest imaging (July 2015 to May 2016). The average age was 68 (range 58 to 84). 8 of the 11 participants consented to, and were able to tolerate, early post treatment imaging. Chemotherapy regimens were either 1^st^ or 2^nd^ line treatments: combinations of oxaliplatin, irinotecan, fluorouracil, folinic acid, capecitabine, bevacizumab, panitumumab. The average time between acquisitions was four days. The average number of days between initiating chemotherapy and post treatment imaging was 5.5 days (range 2 to 11 days). The average number of days between the retest and post-treatment acquisition was 12 days (range 3 to 20 days).

Average mean ADC for 15 delineated tumour ROIs was 121 × 10^−5^ mm^2^/s (A) and 122 × 10^−5^ mm^2^/s (B) which is in line with previous measurements of colorectal liver metastases. Absolute values of mean ADC were consistent before and after motion correction. CoV between A and B was 5.6% (test group) and 7.7% (retest group) (Table 2). Retrospective motion correction did not therefore adversely affect absolute mean ADC values compared to the ROIs delineated from A. CoV for tumour volumes (test A vs. test B, and retest A vs. retest B) were low (<5.6%). Motion correction (B) therefore did not affect volume delineation when compared to A (Table 2).

### Applying the statistical error model for estimation of measurement uncertainty

**Figure 3:**
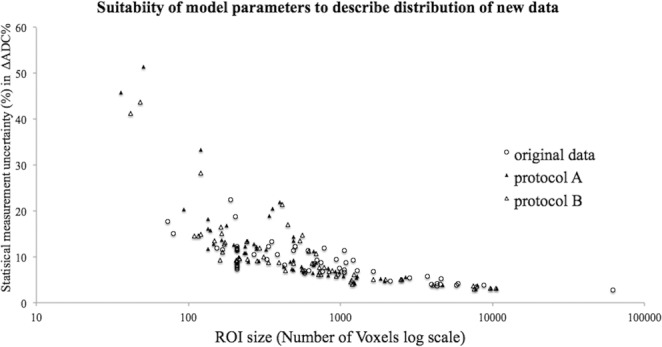
The distribution of uncertainty for both non-motion corrected A (solid triangle) and motion corrected B (triangle) follows a similar inverse relationship with ROI size as previously published quality assured data (no visible motion artefact) (circle), however A data is more scattered.

Figure 3 displays the distribution of statistical measurement uncertainty estimated for each ΔADC% (all defined ROIs), comparing the standard method A and motion corrected method B from this single site data set to the original data set previously published that was used to fit the model (quality assured non-motion affected data). The overall shape of the distribution was consistent, showing an inverse relationship with the number of voxels within an ROI.

The suitability of the model to describe the present data set of non-motion corrected and motion corrected ROIs, was assessed using χ^2^ distributions testing. Table 3 outlines the calculated the χ^2^ statistic (critical chi square value) for each histogram metric. The distribution of uncertainty estimates for ΔADC% measurements using A was different to the model distribution, for all histogram metrics. Eliminating the major contribution of motion (B), improved the fit for almost all metrics, with no difference between the distribution for uncertainty estimates of ΔADC% measurements and the model. As expected, motion was the dominant process that contributes to poor repeatability.

After accounting for motion, the relationship between tumour size and statistical uncertainty in the estimate of ΔADC% could be quantified more precisely. For the 95^th^ percentile, B failed to sufficiently correct for motion, and therefore the model could not be applied to estimate statistical measurement uncertainty. In contrast, B successfully corrected for motion enough that the 5^th^ and 20^th^ percentiles (theoretically the population of voxels with the highest diffusion restriction and tumour density) could be corrected for statistical measurement uncertainty using the error model (S).

Group CoV for A (test-retest) was 9.8% (median ADC), compared to 3.2% for B (test retest).

### Observations of ΔADC% as a marker for early response to treatment

**Figure 4:**
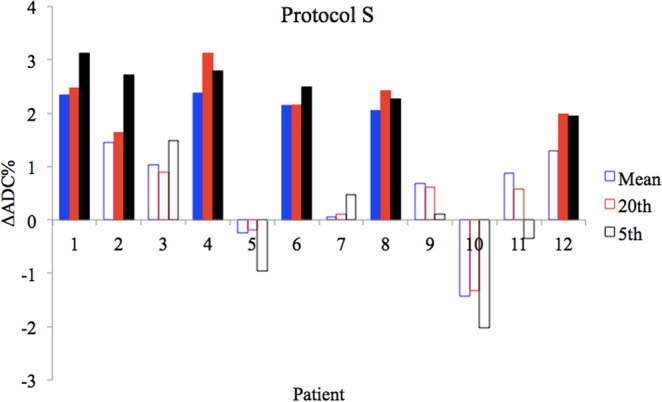
Tumours with statistically significant observed post treatment ΔADC% are highlighted (solid bars). For mean ADC significant ΔADC was observed in 4/12 tumours when motion and statistical error were accounted for (protocol S). For the 20^th^ and 5^th^ percentiles significant ΔADC was observed in 6/12 tumours. For the 5^th^ percentile using significant ΔADC was observed in 6/12 tumours.

ADC histograms were defined for 12 tumours in 8 patients who underwent post treatment imaging. Comparing A, B and then applying the error model (S) for each histogram metric, the 95% LoA was used to determine a statistically significant threshold for changes in ΔADC% (Table 4). After correcting for motion and for statistical uncertainty in the estimated ΔADC% measurement, the lower ADC percentiles were the most sensitive to change, and these changes are highlighted for each post treatment tumour in Fig. 4. The 5^th^ and 20^th^ percentile ΔADC% was statistically significant in 6/12 tumours, compared to 5/12 for the median and 4/12 for the mean. No post treatment tumour ΔADC% value for any histogram metric was significantly less than the lower 95% LoA.

**Table 4:** Comparison of the 95% LoA for ΔADC% between methods.

Histogram	Method A	Method B	Method S
Mean	30.3%	8.7%	1.7%
Median	30.6%	9.1%	1.8%
5^th^ percentile	37.5%	14.8%	2.2%
20^th^ percentile	31.4%	11.9%	1.8%
95^th^ percentile	32.4%	10.7%	—

The 95% limits of agreement (LoA) are used to determine a statistically significant (p < 0.05) percentage change in ADC (ΔADC%). For the 95^th^ percentile, although motion correction improved the threshold for a significant change, the accuracy of any ΔADC% measurement could not be quantified, as the uncertainty model could not be applied (see Table 3).

### Observations of ΔADC% against post treatment LDH trends

**Figure 5:**
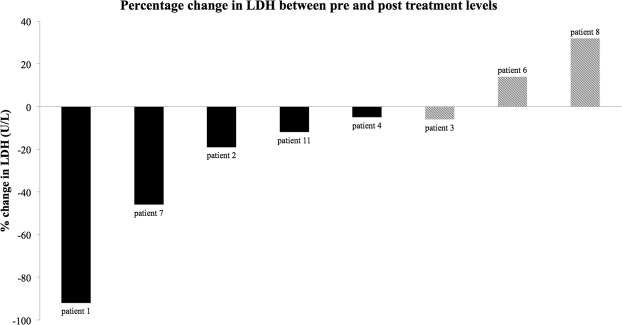
LDH (U/L) levels were collected as part of routine care before treatment started and then every 2 weeks thereafter. ΔLDH% was calculated between the pre-treatment and 3 months post treatment level. The solid black bars indicate patients with tumours that demonstrated significant post treatment ΔADC% after accounting for motion and statistical measurement uncertainty (5^th^ and 20^th^ percentiles). The hatched bars indicate those tumours where there was no significant post treatment response observed for any percentile. Using method S for the 20^th^ percentile data, a Pearson Correlation Coefficient of 0.65 was calculated, with a p value of 0.081. This was not statistically significant at the desired p < 0.05, however with a larger cohort, a correlation between ΔLDH% and ΔADC% may be observed.

Figure 5 is a comparison of ΔLDH% (U/L) from pre-treatment levels to up to 3 months post treatment. Early ΔADC% was seen in 5/6 patients with reducing ΔLDH% and 0/2 where ΔLDH% rose. Using the 20^th^ percentile as an example (6/12 tumours demonstrating a significant ΔADC%) the Pearson Correlation Coefficient between protocol S ΔADC% and ΔLDH% was 0.65 with a p value of 0.081, therefore not significant at the desired level (p < 0.05). Clearly these observations cannot be used to make any claims of correlation on such a small patient cohort, however this demonstrates the potential for combining other biological markers together with an accurate and sensitive ΔADC%, to increase confidence of a post treatment response to therapy.

## Discussion

The results of this study, comparing three alternative methods to detect post treatment changes in colorectal liver metastatic tumour ADC, demonstrates the importance of addressing misregistration caused by respiratory motion. Any method that successfully corrects reduced image quality resulting from motion should be capable of producing a similar improvement to those from this study. If motion correction strategies are not applied then strict quality assurance to exclude degraded images would be required, reducing the number of data sets that can be included in analysis. In this study, motion correction led to at least a 20% improvement of histogram metrics to detecting change (95% LoA) compared to the standard method. With this threshold, at least 4/12 tumours demonstrated early post treatment ΔADC%, where no significant changes were seen previously using the standard approach (A).

In a recent publication of extracranial soft tissue ADC repeatability^14^ 141 lesions from 10 similar studies, were stratified into ROI volumes (smallest third, middle third and largest third), and it was observed that the “large” volume group had a statistically significant smaller CoV (less than 3%) compared to the other groups. This is attributed to increased sample size, reduced motion and less partial volume effect. Based on their findings, a conservative CoV of 6.5% is suggested as a threshold for future studies where repeatability of the group is not possible and tumour volumes are mixed. Using such a threshold would lead to misinterpretation of error as true biological change, for individual tumours, especially for smaller tumours. In small studies such as ours, a sample of 15 tumors does not provide the statistical power needed to observe a meaningful difference in precision (reciprocal variance) between methods A vs. B. This is partly so because many of the tumors are small, hence, yield noisy mean ADC measurements. 15 observations are insufficient to overcome sampling error of a second order statistic (ADC distribution variance).

Combining motion correction with an estimation of the level of statistical uncertainty in the accuracy of ADC estimates is directly inversely proportional to sample size/tumour volume^25^. In this study, correcting for differences in uncertainty between tumours improved estimations of ADC repeatability to within 1.8% for 95% LoA (mean, median) (Table 4). The model cannot be used in isolation with standard protocol image acquisitions that have not been tightly quality assured, as it does not take into consideration motion effects. For this reason, a highly significant disagreement was observed between the model and all histogram metrics for A and 95^th^ percentile for B. Only by combining both complimentary methods, the described repeatability was achievable.

Using the results for mean ADC for S as an example, significant ΔADC% was observed in 4/12 post treatment tumours. One of these was a different lesion to those identified by B. The tumour, which showed significant change with B, but not with S, was small and had a high uncertainty in ΔADC%. After this uncertainty was corrected for, ΔADC% fell below the 95% LoA. Conversely a large tumour ΔADC% became statistically significant only after correcting, as there was less uncertainty in the accuracy of the measurement.

The two latter examples highlight the importance of accounting for uncertainty in the accuracy of a given measurement by scaling to statistical measurement error. Adding 150 extra voxels of data for the small tumour, would have pushed the ΔADC% into a statistically significant observation (for the same measured ADC and distribution width). Motion correction alone would have been insensitive to an observed ΔADC% for the large tumour if consideration were not given to the low level of uncertainty, i.e. increased confidence in the accuracy of the observed estimation.

When both approaches are combined (S) for the lower percentiles (20^th^ and 5^th^), the number of tumours with a statistically significant observed ΔADC% increased from 4 to 6. Despite the relative increased 95% LoA (Table 4), these percentiles were the most sensitive to ΔADC% in the post treatment cohort (Fig. 4). As observed in other studies^16–18^, theoretically this may be related to a larger shift in the ADC histogram at the lower percentiles as intra-tumoural regions with dense populations of malignant cells (higher diffusion restriction and therefore SNR) undergo death and necrosis after treatment.

Ideally, repeatability assessment within a shorter timeframe (24 hours) would have limited any variability due to biological disease progression. The clinical performance status of the recruited volunteers, given the palliative nature of disease, and timing with routine care, limited the flexibility for scanning. Variability in the timings for post treatment acquisitions may have affected the results for repeatability of ΔADC%. An optimal evidence based time for post treatment scanning should be investigated.

ADC distribution width will be affected by precision of the delineation of tumour boundaries, which will impact test-retest repeatability. This error is quantified within the model. We acknowledge however, in the real-word clinical scenario of tumour response assessment, there will be an additional error from delineation of tumour boundaries when different observers (e.g. two different clinicians) assess pre and post treatment tumours separately.

## Conclusion

In this single site study, we have demonstrated that combining retrospective motion correction with an estimation of statistical measurement uncertainty improves estimation of repeatability and increases the likelihood of observing a significant early post treatment response, with a ΔADC% threshold for significant change of 1.7% (mean ADC) in this cohort. Our post treatment ΔADC% results have shown however that the lower percentiles (20^th^ and 5^th^) may be more sensitive to ΔADC% when using this combined method. As Supplementary Data we have provided a spreadsheet that can be expanded and populated with test retest or pre and post treatment ADC data. Provided the number of voxels, ADC and standard deviation of the histogram for each baseline is known, the statistical measurement uncertainty for ΔADC% is automatically calculated, together with a χ^2^ goodness of fit that determines whether the model parameters are suitable to be used for a given data set.

## Supplementary information

Supplementary Information

Statistical Uncertainty calculator

## Data Availability

The full dataset of ADC values for all defined ROIs and subsequent calculations are provided within a protected Microsoft Excel file (Supplementary Data). A table is provided (unprotected) that can be expanded and populated with test retest or pre and post treatment ADC data. Provided the number of voxels, ADC and standard deviation of the histogram for each baseline is known, the statistical measurement uncertainty for ΔADC% is automatically calculated, together with a χ^2^ goodness of fit that determines whether the model parameters are suitable to be used for a given data set.
