# Supplementary material for: Considering tumour volume for motion corrected DWI of colorectal liver metastases increases sensitivity of ADC to detect treatment-induced changes
Source: Sci Rep. 2019 Mar 7;9:3828. doi: 10.1038/s41598-019-40565-y (PMC6405765; doi:10.1038/s41598-019-40565-y)
Supplement: Supplementary file 1 — Supplementary Information [file 41598_2019_40565_MOESM1_ESM.doc]

Considering tumour volume for motion corrected DWI of colorectal liver metastases, increases sensitivity of ADC to detect treatment-induced changes

*Ryan Pathak ^1, 2^, Jingduo Tian ^1^, Neil A Thacker ^1^, David M Morris ^1, 4^, Hossein Ragheb ^1^, Charles Saunders ^3^, Mark Saunders ^2^, Alan Jackson ^1^

^1^University of Manchester, Wolfson Molecular Imaging Centre, Manchester, UK. ^2^The Christie Hospital, NHS Foundation Trust, Manchester, UK. ^3^University of St Andrews, Scotland, UK. ^4^MRC Centre for Inflammation Research, University of Edinburgh, Edinburgh, UK. Correspondence and request for materials should be addresses to RP (email: ryan.pathak@alumni.manchester.ac.uk).

Supplementary Information

**Appendix 1**

Local Rigid Alignment (LRA)

When developing our local rigid alignment (LRA) method we were interested in studying ADC metrics corresponding to a single region in a specific slice of the image. Our LRA method aligns the data corresponding to one slice location, but can potentially also be used for 3D ROIs by aligning adjacent slices separately. Our method involved alignment of a single reference slice to all nearby repeat acquisitions of slice data. The reference slice was taken from the lowest b-value image (b = 100s/mm­^2^), which had the best signal to noise characteristics. The region on which matching was performed was a region covering the tumour. This area contains various boundaries that provided the information needed for matching. We observed that data away from the spine and around the edge of the body generally appeared to move in one (outward) direction. This area was manually identified for the reference slice. Alignment then worked as follows. Using observations of respiratory motion in typical data sets we were able to set limits on the amount of motion present in the region of the liver. We expected no more than 3 pixels movements in vertical and horizontal directions on each image slice and 2 slices along the z-axis. There was little or no observable rotation (less than a pixel at the edge of a ROI), so that movement could be modelled on the basis of shifts of the origin. For each gradient direction there were 6 repeat ‘volume’ acquisitions. We allowed 5 adjacent slices per repeat data as candidates for alignment, the central slice and two slices from either side. Hence for each gradient direction there were 30 candidate slices from which 6 slices were selected with best matching scores.

We developed our own matching algorithm for a reference slice against a target slice based upon conventional statistics. Applying the variational principle to the problem of matching two scaled noisy image patches *I* and J (with *N* pixels and standard deviation of image noise *σ*), one can define the optimisation function [4]


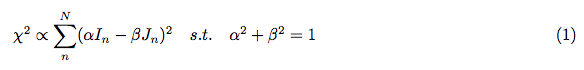


where *χ* ^2^ is proportional (and not equal) to the term shown on the right-hand side (which theoretically suffice for optimisation). However, we can explicitly derive formulations and rewrite the cost function in form of detailed equations as follows. Specifically, rather than using two scale factors *α* and *β*, one may use a single scale factor *γ = α*/*β*. For similar patches it can be shown that
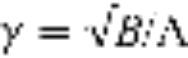
 where


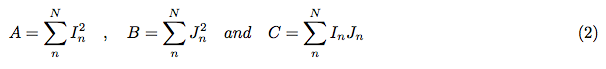


To avoid lengthy execution times, one may expand the patch similarity measure and write


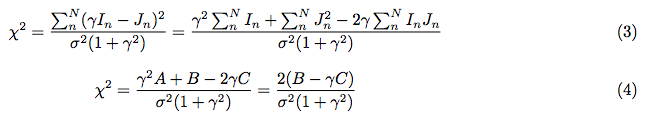


When choosing the reference image patch to be *J*, it follows that *B* is the constant term while *A* is varying as the target image patch is translated. Also *σ* has a fixed value during optimisation. Hence minimising *χ* ^2^ in Eq. (4) is equivalent to minimising


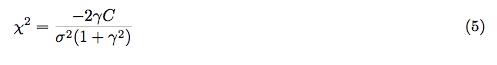


The γ/(1 + γ ^2^) term can be eliminated during optimisation in accordance with the general proof of convergence of the Expectation Maximisation (EM) algorithm [^1^](#_ENREF_1), so that optimising the term C is equivalent to optimising the χ ^2^ . The term C works well when used in a conventional optimiser for alignment of similar images. However, here rather than using grey-level image patches and least-square differences directly, their gradients (based on differential kernels) in the horizontal and vertical directions are used. Previous work [^2^](#_ENREF_2) in medical image analysis and machine vision has demonstrated that this reduces the dependency upon absolute scaling of the data and is more suitable for matching with MRI and CT datasets, though at the expense of reducing the capture range of the cost-function. In this case, C can be interpreted as the summation of the ‘dot products’ of the two-component gradient vectors originated from the reference and target image patches. As we do not need to consider sub-pixel shifting, we exhaustively compute the cost function for the limited number of possible variations and find the values, which give the best matching score to avoid local optima. Among our candidate patches there will be sub-pixel shifted versions of the region (due to the anatomical motion). As we select only the best matching images from those available we therefore recover some amount of sub-pixel precision whilst avoiding interpolation smoothing.

1 Dempster, A. P., Laird, N. M. & Rubin, D. B. Maximum Likelihood from Incomplete Data via the EM Algorithm. *Journal of the Royal Statistical Society. Series B (Methodological)* **39**, 1-38 (1977).

2 Bromiley, P. A., Schunke, A. C., Ragheb, H., Thacker, N. A. & Tautz, D. Semi-automatic landmark point annotation for geometric morphometrics. *Frontiers in Zoology* **11**, 61, doi:10.1186/s12983-014-0061-1 (2014).

**Appendix 2**

The ADC is the decay parameter from an exponential fit of the loss in signal intensity at a given pixel location, between the increasingly diffusion sensitive images (b-100, 500, 900 s/mm^2^). In clinical data, as the noise distribution is skewed towards the higher b-values, we find that a first order bias correction factor ($\alpha$) improves the quality of fit as it removes the SNR dependent bias.

We estimate ADC (referred to as *D*), through a likelihood-based parameter optimization, log *P*(*I\D*, *S*0) (the probability of the image data given the assumed parameters)

$$\log P\left(I|D,S0\right)=\frac{-1}{2\sigma^{2}}\sum_{b_{k}} [I(b_{k})-f(b_{k},D,S0)]^{2}+const$$

where $f(b_{k},D,S0)]^{2}$is the theoretical value of the bias corrected exponential function and $I(b_{k})$is the signal value from the b-value image pixel. $f$is a function of b-value $b_{k}$ and the current estimates of ADC, *D*, and no-diffusion signal, *S*0 (at $b_{k}$= 0). This is computed using

Where $k\in\left[1,2,3\right]$ refers to the three b-values $b_{k}$ used, e.g. 100, 500 and 900 s/mm^2^. The signal value for no diffusion (b=0) *S*0 is the second parameter that is estimated. $\alpha$is a fixed value defining the amount of bias correction applied and it may be adjusted depending on the amount of image smoothing corresponding to the specific imaging protocol used by the scanner (for our data $\alpha$was set to the theoretical value of one). An estimate of the standard deviation (SD) of noise in the image
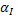
 is computed from the distribution of second derivatives (for x and y) around zero, in a central rectangular region on the tissue (<http://www.tina-vision.net/docs/memos/2008-010.pdf>).
